# Does Human Touch Facilitate Object Categorization in 6-to-9-Month-Old Infants?

**DOI:** 10.3390/brainsci10120940

**Published:** 2020-12-06

**Authors:** Girija Kadlaskar, Sandra Waxman, Amanda Seidl

**Affiliations:** 1 Department of Speech, Language, and Hearing Sciences, Purdue University, West Lafayette, IN 47906, USA; aseidl@purdue.edu; 2 Department of Psychology, Northwestern University, Evanston, IL 60208, USA; s-waxman@northwestern.edu

**Keywords:** infants, categorization, touch, communication, non-speech

## Abstract

Infants form object categories in the first months of life. By 3 months and throughout the first year, successful categorization varies as a function of the acoustic information presented in conjunction with category members. Here we ask whether tactile information, delivered in conjunction with category members, also promotes categorization. Six- to 9-month-olds participated in an object categorization task in either a touch-cue or no-cue condition. For infants in the touch-cue condition, familiarization images were accompanied by precisely-timed light touches from their caregivers; infants in the no-cue condition saw the same images but received no touches. Only infants in the touch-cue condition formed categories. This provides the first evidence that touch may play a role in supporting infants’ object categorization.

## 1. Introduction

The way we categorize objects impacts how we perceive relationships among them [1,2,3]. Without categorization, we would treat every entity we encounter (e.g., every golden retriever) as a unique individual. Identifying a category to which an object or entity belongs (e.g., a dog, a retriever) permits us to make inferences that go beyond our immediate experience with that individual (e.g., that retrievers bark, have sharp teeth, etc.). Categorization, therefore, is a fundamental cognitive process, and one that is especially important for young infants who encounter new objects, entities, and events every day. A considerable body of research reveals that in the first months of life, infants successfully and spontaneously form object categories, including visual object categories of animals, vehicles, and geometric shapes [4,5,6,7,8,9,10].

Moreover, for infants as young as three months of age, object categorization is boosted when visual images of category members (e.g., dinosaurs) are presented in conjunction with human speech, but not when the same visual images are accompanied by other auditory signals, including backward speech and pure sine wave tone sequences [4,8,9,11,12,13]. Thus, listening to human speech promotes object categorization in infants, in a way that listening to other well-matched non-speech auditory stimuli does not.

It is possible that for very young infants, who have not yet begun to produce language on their own, human speech promotes categorization, at least in part, by virtue of its social-communicative status. To test the power of communicative status on object categorization, Ferguson and Waxman [11] systematically manipulated the communicative status of sine wave tone sequences. To do so, they first presented infants with a video of two actors engaged in an animated dialogue for 2 min, one speaking and the other responding in (dubbed) tone sequences. Embedding the sine wave tones, an otherwise inert signal, within the context of this rich communicative episode had a striking effect: in a subsequent task, infants who had viewed this communicative dialogue went on to form object categories successfully. Their success stands in sharp contrast to the infants who also viewed a video in which the same tone sequences were presented in a non-communicative context [11]. This outcome suggests that signals other than human speech can promote learning under certain conditions. What remains unknown is the breadth of signals that might support this cognitive advantage in young infants. Thus far, experimental work has focused exclusively on signals presented in the auditory and visual modalities.

Here, we move beyond auditory and visual signals to consider the contribution of caregivers’ tactile input—a ubiquitous stimulus that is available to infants from birth. The sense of touch, which develops early, is an essential point of contact with the external world for young infants [14,15]. Indeed, there is evidence that touch may impact infants’ early development [16,17] by promoting parent–infant bonding [18] and secure attachment [19], and by reducing infant distress [20]. While it is not clear whether infants in the first year of life perceive the tactile input they receive as communicative, recent evidence suggested that caregiver touch promotes infant speech perception [21], abstract pattern learning [22], and lexical acquisition. For example, infants’ early vocabularies tend to include words that are frequently used in conjunction with caregiver touches [23,24]. Thus, it is possible that tactile input may heighten infants’ attention to concurrent stimuli present in their surroundings, including the objects, events, and interpersonal interactions which may provide a foundation for infants’ learning. In sum, touch is not only ubiquitous in infant-caregiver interactions but also appears to support infant development and learning [25].

Therefore, there is reason to suspect that human touch, when paired with visual images, might boost object categorization. We test this hypothesis, focusing on the contribution of touch to 6- to 9-month-old infants’ object categorization in two conditions: a touch-cue condition and a no-cue condition. For infants in the touch-cue condition, visual familiarization stimuli are presented in conjunction with precisely-timed gentle touches, presented on their knee by caregivers; infants in the no-cue condition viewed the same images, but these were not accompanied by caregiver touch (Our decision to have mothers touch their infants on the knee is built upon on evidence that 4-month-olds can use touch delivered on their knee or elbow as a cue to word boundaries [21] and as a cue to abstract pattern learning [22]. For example, Seidl and colleagues showed that the presentation of consistent tactile stimuli (on the knee or elbow) that is aligned with speech facilitated infants’ success in finding specific units in the continuous stream of speech. In addition, parents report that delivering the touches on their infants’ knees is natural and convenient when an infant is seated on their lap. Finally, Abu-Zhaya et al. [23] report that caregivers naturally and spontaneously touch their infants on the knee during joint book reading. For these reasons, tactile stimuli in the present study was delivered on the infant’s knee.). Our decision to focus on infants at 6 to 9 months of age is motivated by evidence that caregivers provide considerable tactile input in the form of interpersonal touches at this age [26] and by evidence that even certain other non-speech signals support object categorization at this age [11].

## 2. Materials and Methods

Six- to 9-month-olds participated in a frequently used object categorization task [4,8,9,12]. During the familiarization phase, infants viewed eight different images, presented one at a time, all from the same category (e.g., a variety of dinosaurs). At test, infants viewed two new images presented side-by-side in silence, one from the now-familiar category (e.g., another dinosaur) and the other from a novel category (e.g., a fish). Infants’ looking preferences at test were computed by dividing total looking time to the novel test image by total looking time to the novel and familiar test images combined. The resulting novelty preference scores range from 0 to 1, and a reliable preference for one test image over the other (i.e., significant difference from chance; 50%) is taken as an index of successful categorization [6,9]. In a typical categorization task, this novelty preference score is used to determine whether infants prefer a novel image (i.e., looking longer at the fish when familiarized with dinosaurs) or a familiar image (i.e., looking longer at the dinosaur when familiarized with dinosaurs) as compared to no significant preference towards either of the stimuli.

### 2.1. Participants

Participants included 54 (27 female) healthy, full-term hearing infants between 6.5 and 9 months of age (M = 7.69 months, SD = 0.76). Half of the infants (*n* = 27) participated in the touch-cue condition and the other half (*n* = 27) participated in the no-cue condition. Infants were recruited from birth announcements and/or social media. A total of 50 infants were from monolingual English-speaking homes; four (one in the touch-cue condition and three in the no-cue condition) were from non-English-speaking homes (note that we did not expect home language to be a factor given that no speech was presented in our task). Data from 13 additional infants were excluded because of fussiness (6), experimenter error (4), and looking to the screen for less than 25% of familiarization (3).

### 2.2. Stimuli

#### 2.2.1. Visual Stimuli

**Figure 1:**
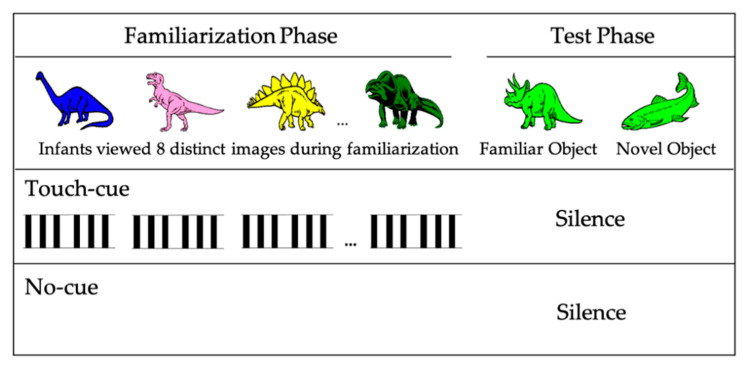
Representative set of visual and tactile stimuli for familiarization and test trials. Black bars in the touch-cue condition represent. 0.45 s touches delivered to the infants.

Visual stimuli were identical to those used in prior work [4,8,9,12]. These consisted of line-drawn images of dinosaurs and fish (Figure 1) presented during familiarization and test.

#### 2.2.2. Tactile Stimuli (Presented in the Touch-Cue Condition Only)

Only one variable was manipulated between the touch-cue and the no-cue conditions. Specifically, during the familiarization phase of the touch-cue condition, visual images from a category (e.g., dinosaurs) were accompanied by precisely-timed tactile cues provided by caregivers. The familiarization phase in the no-cue condition was similar except that infants did not receive any touches by their caregivers. No touches were delivered in either condition during the test phase of the experiment.

In what follows, we detail caregivers’ training and delivery of touches during the familiarization phase in the touch-cue condition.

Caregiver training: Before the experiment, infants assigned randomly to the touch-cue condition played with a lab assistant while an experimenter trained caregivers to gently squeeze their infant’s knee when they heard an auditory cue (a 300 Hz sinewave touch-cue tone), presented over headphones. All caregivers were instructed to move their hand away from their infant’s knee after each touch. Next, caregivers were instructed to deliver 48 precisely-timed touches to their own knee as practice; all caregivers successfully delivered at least 46 touches in response to the touch-cue tones during training. Caregivers in the no-cue condition received no training; they were instructed only to support their infants on their laps.

Tactile cues during the experiment: As they had during training, during familiarization, caregivers in the touch-cue condition heard a series of 48 tones presented over headphones, each lasting 0.45 s (Figure 1). A verbal command presented before the first familiarization trial alerted caregivers that touch-cue tones were about to begin. Each tone was preceded by a brief alerting tone to signal that a touch-cue tone was coming. During each visual familiarization trial, touches were delivered twice: in the first 4 s and again 3 s later. This mirrors the timing of presentation of auditory cues in prior work using this paradigm [4,8,9,11,12]. At the end of the last familiarization trial, caregivers heard a verbal prompt over headphones that marked the end of caregiver touches and familiarization.

### 2.3. Apparatus

Infants sat on their caregivers’ laps, approximately 185 cm away from a 124 × 29 cm visual monitor. Infant eye gaze and caregiver touches were recorded from a video camera hidden in a panel below the monitor. Peltor headphones were provided to caregivers during training and familiarization to hear touch-cue tone sequences.

### 2.4. Procedure

Infants visited the lab with their caregivers. An experimenter explained the procedure and caregivers of all infants provided written informed consent. After this point, caregivers in the touch-cue condition received training on how and when to touch their infants during familiarization (above) while their infants played with an experimenter.

During the experiment proper, each infant was seated on their caregiver’s lap. During familiarization, all infants viewed eight images, each featuring a different exemplar from the same object category (infants were randomly assigned to either the dinosaur or the fish group, with half of the infants in each group) that appeared in a random order one at a time (for 20 s each). In the touch-cue condition, infants received touch-cues as described in the stimuli section above. The procedure in the no-cue condition was identical, except that infants did not receive timed touches from caregivers.

After familiarization, infants in both conditions participated in a test involving two new images, presented side-by-side in silence: one, a new member from the now-familiar category and the other, a new member of a novel category. No touches were delivered at test in either condition. The test trial concluded when an infant had accumulated 10 s of looking at the screen. Infants received a book or toy as a token of appreciation for their participation.

### 2.5. Coding Caregiver Touches

A trained research assistant coded the number of touches presented to infants in a frame-by-frame analysis in ELAN (EUDICO Linguistic Annotator; EUDICO stands for European Distributed Corpora Project; Nijmegen, Netherlands) [27]. All caregivers delivered between 46 and 49 touches (in response to the 48 touch-cue prompts). No dyads were excluded from analyses based on the number of touches caregivers provided to infants.

### 2.6. Coding Infant Eyegaze

A trained research assistant, blind to the hypothesis, coded infants’ visual attention in Supercoder [28]. Another assistant, also blind, recoded 20% of the data. Reliability between coders was high (*r* = 0.97 and *r* = 0.98, in familiarization and test phases, respectively).

### 2.7. Ethical Considerations

The study was conducted in accordance with the Declaration of Helsinki and was approved by the Institutional Review Board of Purdue University (0503001761). Written informed consent was obtained from the parents of each infant participant before they participated in the study.

## 3. Results

### 3.1. Familiarization Phase

There were marginally significant differences in looking times between the touch-cue (M = 81.19 s, SD = 23.76 s), and no-cue (M = 69.87 s, SD = 19.43 s) conditions, *t*(52) = 1.91, *p* = 0.06, d = 0.52. In order to examine whether any differences in visual attention to familiarization images were correlated with differences at test, we ran Pearson correlations between individual infants’ looking times during familiarization and their performance at test (measured by novelty preference score; see below) using Statistical Package for Social Sciences (SPSS) software version 26 (IBM Corporation, Armonk, NY, USA). This revealed that Looking time during familiarization was not significantly correlated with performance at test *r*(54) = −0.10, *p* = 0.47. Next, we ran the same correlation for infants in the touch-cue condition only. Again, there was no correlation between infants’ looking times during familiarization and performance at test, *r*(27) = −0.01, *p* = 0.93. These results suggest that any differences in infants’ performance at test cannot be attributed to the amount of time spent looking at the images during familiarization.

### 3.2. Test Phase

We calculated a novelty preference score for each infant (looking time to the novel test image/looking time to the novel and familiar test images combined). Novelty preference score ranges from 0 to 1 and a reliable preference for one test image over the other (i.e., significant difference from chance; 50%) are taken as an index of successful categorization [6,9]. Using one-sample *t*-tests, we first examined whether infants in either the touch-cue or no-cue conditions revealed novelty preferences greater than would be expected by chance (50%). Infants in the touch-cue condition revealed a significant preference for the novel test image, (M = 0.55, SD = 0.13), *t*(26) = 2.07, *p* = 0.04, *d* = 0.44. In contrast, those in the no-cue condition performed at chance, (M = 0.53, SD = 0.17), *t*(26) = 1.16, *p* = 0.25, *d* = 0.22. According to Cohen’s guidelines, the effect size was medium in the touch-cue condition and small in the no-cue condition.

In order to supplement our one-sample *t*-tests analysis, we analyzed individual infants’ responses to assess whether success in the touch-cue condition as a group is characteristic of most infants in this group. Following previous research with this categorization paradigm [9], we tallied the number of infants in each condition who showed a novelty-preference (>0.50). In the touch-cue condition, 70% of the infants (19/27) showed a novelty-preference (*p* = 0.05, two-tailed). In contrast, in the no-cue condition only 52% (14/27) showed a novelty-preference (*p* = 1, two-tailed). This outcome, coupled with the one-sample t-test analyses, suggests that touch may play a role in supporting infant object categorization.

Next, to identify any interactions between Condition and Test Looking Time, and to assess whether this varied as a function of infant age, we performed an Analysis of Covariance with Condition (touch-cue, no-cue) as a between-subjects factor, Test Looking Times (looking to novel, looking to familiar) as a within-subjects factor, and age as a covariate. Test Looking Times for novel and familiar images were measured by examining infants’ total looking times to novel and familiar images during the test phase. Results revealed no interaction between Condition and Test Looking Times *F*(1,51) = 0.02, *p* = 0.87 while controlling for age and no main effects of Condition *F*(1,51) = 1.59, *p* = 0.421 or Test Looking Times *F*(1,51) = 0.95, *p* = 0.33. Finally, we also analyzed our results using Bayesian statistics to examine group differences. Bayesian statistics were included because Bayes Factor values indicate the strength of evidence in favor of both the null and alternate hypotheses. Bayesian analysis confirmed that test performance in the two conditions did not differ significantly (Bayes Factor = 0.11; interpreted as substantial evidence in favor of the null hypothesis).

## 4. Discussion

For infants at 6 to 9 months of age, presenting visual images systematically timed in conjunction with a tactile signal may provide some support in forming object categories. This provides the first indication that the cognitive advantages conferred by certain acoustic signals may extend to include tactile cues. Infants exhibited a significant novelty preference in the touch-cue (but not the no-cue) condition when compared against chance. This outcome, consistent with evidence that caregivers’ tactile cues facilitate processing of accompanying auditory stimuli [21,22], suggests that tactile cues may also facilitate infants’ processing of accompanying visual stimuli. We argue that infants’ performance at test may be mediated by their ability to learn the meaningful associations between tactile cues and visual images. The current results offer additional insight into the utility of tactile cues in infancy when caregiver touch is ubiquitous [17,18,19,20]. However, our findings must be interpreted with caution: Infants successfully formed object categories in the touch-cue (but not the no-cue) condition when compared against chance, but the difference between these conditions at test was not statistically significant. In future work, therefore, it will be essential to replicate and broaden these findings.

It will also be important to consider the mechanism(s) by which certain non-speech signals (including touch) exert advantageous effects on infant cognition, should this effect hold up on replication and extension. We suspect that maternal touches during familiarization may have augmented infants’ attention to the familiarization images. Infants’ attention to the visual images may be mediated by many factors. For example, some have argued that low-level attentional mechanisms provide a sufficient account for the cognitive advantages conferred by listening to language. More specifically, the claim is that infants’ successful object categorization when listening to language and their chance-level performance when listening to non-linguistic acoustic signals (e.g., tone sequences) reflects their familiarity with the acoustic signals: because infants are more familiar with the sounds of speech than with other non-linguistic sounds [29,30,31,32], and because processing familiar stimuli is less costly than processing unfamiliar ones, the claim is that the less familiar signals (e.g., tone sequences) detract from infants’ processing of the accompanying visual materials (also see [33,34]). In this view, listening to language is merely less disruptive than listening to tone sequences. There are several reasons to doubt this interpretation, chief among them is the evidence that infants categorize successfully when listening to sounds as unfamiliar as the vocalizations of non-human primates [12,35]. The evidence reported here, documenting infants’ performance in silent conditions (both the touch-cue and no-cue conditions) in which auditory overshadowing could not occur, also presents a challenge to his interpretation. Infants’ performance in the touch-cue condition, considered in conjunction with infants’ success in previous studies while listening to language [8,9,12,36], communicative tones [11], and non-human primate vocalizations [12,35], rules out auditory overshadowing as a descriptive or explanatory account.

The current study is not without limitations. First, infants in the touch-cue condition received touch cues that involved a combination of visual and tactile input: caregivers’ hand movements while delivering touches to their infants’ knees. Although it is unlikely that viewing touches aids object categorization (see [21] for lack of robust results in statistical learning with viewed touches), providing touch in synchrony with the visual information may have resulted in more robust attention than touch alone. In future work, it will be interesting to assess whether touch alone supports infant categorization or whether perceiving touch visually and haptically, in combination, is required.

Second, infants in the touch-cue condition spent a marginally greater amount of time looking at the familiarization images compared to infants in the no-cue condition, raising the question of whether it was greater amounts of attention/looking times during familiarization in the touch-cue condition that impacted our results. While our correlational analysis suggested that infants’ looking times during familiarization were not related to performance at test, further research is required to systematically examine the role of attention on object categorization. Third, we acknowledge that infants in the touch-cue condition spent about 5 min more in the lab with an examiner while their caregivers completed the touch-cue training than did infants in the no-cue condition whose experimental session began right after the consenting procedures. However, given that this additional time is within the typical window of time that infants usually spend in the lab during experiments (in both conditions), we do not think this impacted looking patterns. Furthermore, there is no precedent from previous studies of this design that involves tracking the impact of time spent in the lab before an experiment (e.g., with caregivers chatting with an experimenter) and experiment outcomes, though we know anecdotally that this varies with each family visit. Last, although the current study offers preliminary evidence that touch, when paired systematically with images, may support object categorization, it remains unknown whether this advantage relies upon the synchronous pairing of touch and image, or whether it might also be evident with less systematic occurrences of touch.

Finally, our results have implications for understanding the role of touch in early learning. We know that listening to native speech facilitates objects categorization in infants as young as 3 months of age [8] and that this advantageous effect is evident throughout the first year [4,9]. The present study adds to this literature by showing that, like speech, interpersonal touch may also have an advantageous effect on cognition in infancy. This possibility is important, especially considering the ubiquity and frequency of touch during infants’ early social interactions [20,37] and the richness of interpersonal tactile interactions (e.g., caregivers often combine relevant tactile and speech cues while communicating with their infants; [23]). Our findings also support extant evidence of the function of touch in infants’ early learning [21,22]. Specifically, past research has shown that tactile information, provided synchronously with speech, supports typically developing infants’ success in finding word boundaries [21] and in learning abstract auditory patterns [22]. Although in the present study we did not examine the role of touch as it relates to speech processing, we did show that touch may facilitate the formation of object categories, a fundamental cognitive process that supports, and is supported by, language [38]. Because touch has been shown to facilitate speech perception [21], rule learning [22], and object categorization (present study), future research should examine the role of touch as it relates to speech perception in the context of object categorization.

## 5. Conclusions

The results reported here offer support for the hypothesis that touch, like language, may facilitate object categorization in 6- to 9-month-old infants. To the best of our knowledge, this is the first study to examine the role of human touch in infant object categorization. Our study provides the first evidence that acoustic signals may not be the only ones to boost infant categorization in young infants.
